# The comparative importance for optimal climate policy of discounting, inequalities and catastrophes

**DOI:** 10.1007/s10584-017-2094-x

**Published:** 2017-10-28

**Authors:** Mark Budolfson, Francis Dennig, Marc Fleurbaey, Asher Siebert, Robert H. Socolow

**Affiliations:** 1 University of Vermont, Burlington, VT USA; 2 Yale-NUS College, Singapore, Singapore; 3 Princeton WWS-UCHV, CFI, Princeton, NJ USA; 4 Columbia University EI-IRI, New York, NY USA; 5 Princeton CFI, CMI, MAE, Princeton, NJ USA

## Abstract

Integrated assessment models (IAMs) of climate and the economy provide estimates of the social cost of carbon and inform climate policy. With the Nested Inequalities Climate Economy model (NICE) (Dennig et al. PNAS 112:15,827–15,832, 2015), which is based on Nordhaus’s Regional Integrated Model of Climate and the Economy (RICE), but also includes inequalities within regions, we investigate the comparative importance of several factors—namely, time preference, inequality aversion, intraregional inequalities in the distribution of both damage and mitigation cost and the damage function. We do so by computing optimal carbon price trajectories that arise from the wide variety of combinations that are possible given the prevailing range of disagreement over each factor. This provides answers to a number of questions, including Thomas Schelling’s conjecture that properly accounting for inequalities could lead the inequality aversion parameter to have an effect opposite to what is suggested by the Ramsey equation.

**Electronic supplementary material:**

The online version of this article (10.1007/s10584-017-2094-x) contains supplementary material, which is available to authorized users.

## Introduction

Models of optimal economic responses to climate change are contingent on highly uncertain estimates, including about the future societies with which climate will interact. Nevertheless, incomplete estimates exist, and when put into integrated assessment models (IAMs), they serve to provide some guidance on the extent of effort society should dedicate to this problem (Stern 2006; Nordhaus 2010; Nordhaus and Sztorc 2013; Tol 1996, 2009).

There is no consensus that a high mitigation effort is warranted, but there are several instances of such recommendations by researchers using these models—most notably the Stern Review, which computes a high social cost of carbon based on very low discount rates (Stern 2006; Hope 2008; Nordhaus 2007). Other researchers provide a similar sense of urgency based on other features, such as the possibility of catastrophic damages (Weitzman 2009, 2012; Dietz and Stern 2015), the possibility that climate damages will disproportionately harm the poor within countries (Dennig et al. 2015), price effects (Sterner and Persson 2008) and damages affecting growth rates (Moore and Diaz 2015; Dietz and Stern 2015). Ackerman and Stanton (2012) combine variations about the discount rate, climate sensitivity and damages and obtain a wide range of carbon prices with the DICE model.

In this paper, we investigate the interactions between several of these individually important factors by providing a systematic study of the comparative importance for optimal mitigation of inequality aversion, time preference, catastrophic damages and intragenerational distribution of damages and abatement costs. While a longer list of factors could be drawn, this selection enables us to make two main points. First, the direction by which inequality aversion influences optimal mitigation depends on the distribution of mitigation cost and damages within countries and the time preference parameter. Second, the distribution of mitigation costs and damages is of prime importance for optimal mitigation, whereas catastrophes become relevant only when inequalities are a low concern. Our investigation of these issues is made possible by further development of the Nested Inequalities Climate Economy (NICE) model (Dennig et al. 2015), which is based on Nordhaus’s Regional Integrated Model of Climate and the Economy (RICE 2010) (Nordhaus 2010), but also includes mechanisms to represent inequalities within regions and countries that are not included in RICE or in other existing climate-economy IAMs.

Our first point about the importance of the distribution of damages and mitigation cost relates to Thomas Schelling’s conjecture that properly accounting for inequalities within generations could lead the inequality aversion parameter to have an effect opposite to what is suggested by the Ramsey equation (Schelling 1995).^1^ In the paper, we elaborate this conjecture and explain how NICE allows us to evaluate it. We then show that this *Schelling Reversal* can indeed occur but only under particular conditions combining regressive distribution of damages or progressive distribution of abatement costs, and sufficient discounting. The first two conditions result in the poorest in the future being overwhelming beneficiaries of mitigation, while the poorest today are shielded from the lion’s share of mitigation cost. But even in such cases, if there is high discounting, there are still large climate damages in the future. As a result, under those conditions, as inequality aversion increases, optimal mitigation increases to protect the future poor from those damages. Under low discounting, there is very high mitigation, to the point where most damages are avoided. As a result, with low discounting, as inequality aversion increases, optimal mitigation decreases to protect the present (relatively poor) generation, as implied by the Ramsey equation. At intermediate levels of discounting, these two effects balance out, and inequality aversion has a small effect on optimal carbon prices.

After illustrating the importance for optimal mitigation of within-region inequalities and the interaction of mitigation costs and damages with this distribution, we contrast it to the effect catastrophic aggregate damages on optimal mitigation. We compare the RICE model’s quadratic damage functions with specifications with convexities that wipe out half of GDP respectively at 6 °C and at 4 °C of warming above pre-industrial levels. The former is the damage function Weitzman (2012) proposes to model low probability extreme events. We consider these extreme damages *under certainty.* We find that even if such damages occur with certainty, the difference in optimal prices across the three specifications is smaller than for varying the sub-regional distribution of mitigation cost or damage over a credible range. This is the case, as long as the inequality aversion parameter is greater than unity, on which most such studies agree (see Evans (2005) for estimates from OECD countries and Dasgupta (2008) for a theoretical overview). In fact, when the distribution of damage hurts the poor, the specifications with catastrophic damage barely affect the optimal price at all. This is because an unequal damage distribution induces a social catastrophe by hurting the poor, which is optimally avoided. In avoiding it, the high temperatures at which the aggregate catastrophe kicks in are avoided as well, making the high damage at those temperatures irrelevant to the optimum.

Inequalities in the distribution of damage and mitigation cost have already been studied in the IAM literature, but in a less fine-grained way. Shortly after Schelling stated his conjecture, Azar and Sterner (1996) considered the first inequality averse climate-economy IAM. Fankhauser et al. (1997) perform a similar analysis in a multi-region model. Tol (2001) calculates optimal carbon prices in the Climate Framework for Uncertainty, Negotiation and Distribution (FUND) model with an inequality averse welfare function. Hope (2008) uses equity weights in the PAGE2002 model to calculate the social cost of carbon. Anthoff et al. (2009) apply equity weighting to the FUND model to calculate the social cost of carbon for a variety of scenarios. They take account of intercountry inequalities within the regions of FUND, assuming that regional damages are distributed among countries in proportion to population. Dennig (2013) implements RICE without Negishi weights and compares the results for two different social objectives, one with and one without spatial inequality aversion. Anthoff and Emmerling (2016) do the same in the FUND model, introducing intercountry inequalities.

In contrast, this paper relies on a model with inequalities within regions and countries in which the distribution of mitigation cost and damages can be varied, enabling us in particular to study the Schelling Reversal and the relative importance of catastrophes and distributional catastrophes as described above.

## The Schelling Reversal and NICE

In spite of the arguments for urgent mitigation noted in the previous section, there remains a persistent view among some economists that there is scant cause for concern. For example, a meta-analysis of the literature on climate damages (Tol 2009) estimates an economic damage of 0.7% of GDP at 2.5 °C warming. If this is read against a background of growth of GDP at more than 1% per year, it may seem that worrying about the future generations impacted by climate change is not worth the effort—akin to asking Mexico to transfer resources to help a drought-stricken California.

More generally, the growth rate assumed in leading IAMs generates substantial inequalities between generations, as the assumed growth rates and damage functions imply that future generations will grow richer and richer even under business as usual (Dennig et al. 2015; Anthoff et al. 2009). As a result, the optimal amount of mitigation effort depends very much on two parameters in the social objective of these models: first, the rate at which future generations are discounted simply because they are in the future, which is represented by the *rate of pure time preference ρ* and, second, the relative priority of the poor and the rich, which is represented by the *inequality aversion* parameter *η* (i.e. the consumption elasticity of marginal utility). These parameters are grounded by some authors on ethical considerations and by others on observed savings rates coupled with very strong assumptions about savings behaviour. But the common result suggested by the Ramsey equation is that increasing either one of these parameters delays mitigation. That is because the Ramsey equation states that the discount rate on costs and benefits (as measured in dollars) is given by

$$ \mathrm{discount}\  \mathrm{rate}=\rho +\eta \times g $$

where *g* is the average growth rate of consumption between now and the discounted period. This equation suggests that a greater value for either *ρ* or *η* will raise the discount rate on consumption and therefore delay mitigation, given the positive growth rate assumed by these models.^2^

However, if inequalities within generations were taken into account, it is no longer obvious that increasing inequality aversion must have the effect of delaying mitigation that is suggested by the Ramsey equation and commonly assumed in the literature. In particular, Schelling (1995, p. 400) provocatively noted that “once we disaggregate the world’s population by income level, it becomes logically absurd to ignore present needs and concentrate on the later decades of the coming century,” but also that mitigation policy amounts to implementing “transfers [that] will be from the currently rich to the descendants of the currently poor, who will, when the benefits begin to be felt, be much less poor than they are now but still poorer than the descendants of the currently rich and probably still significantly poorer than the abatement-financing countries are now” (p. 399). So, on the one hand, properly representing inequalities reinforces the salience of current needs and may recommend a shift of focus from mitigation to adaptation. But the second quote suggests that an important factor that pulls in the opposite direction is the relative wealth of those who pay for the effort and those who benefit from a preserved climate.

Schelling’s remarks suggest a pressing question: *in spite of the presumption that future generations will be on average richer than the present generation, could it be true that the more one dislikes inequalities and cares for the disadvantaged, the more mitigation one should want to see*? In other words, *in spite of the fact that aggregate growth together with the Ramsey equation suggests that increasing inequality aversion would delay mitigation, could it be true that when inequalities are properly accounted for, increasing inequality aversion actually implies faster mitigation?*

In order to evaluate Schelling’s conjecture that the answer to these questions might be different than suggested by the Ramsey equation, as well to answer other pressing questions about climate and inequality, we have developed a modification of the widely used RICE model, which we call NICE model. As we describe in detail below, NICE incorporates a fuller description of inequalities by income quintile in the distribution of income, damages and mitigation cost within each global region. Schelling’s ideas could be studied with RICE, since inequalities between regions are important and may potentially influence the impact of changing the inequality aversion parameter on the optimal carbon price. However, such inequalities are only one part of the global inequalities within generations, and a fuller study is possible with a more comprehensive depiction of inequalities. With NICE, as explained below, we can actually analyse the possibility of the Schelling Reversal when focusing on inequalities between regions and identify whether inequalities within regions are a crucial element in the reversal as well.

A more detailed description of the model is provided in the online supplement. Here, we describe the essential features necessary to understand the following discussion. The social welfare function is a time-discounted and separable constant elasticity function with population weights:

1 $$ W\left({c}_{ijt}\right)={\sum}_{ijt}\frac{L_{ijt}}{{\left(1+\rho \right)}^t}\frac{c_{ijt}^{1-\eta }}{1-\eta } $$

where *W* denotes social welfare, *L* population, *c* per capita consumption, *ρ* the rate of pure time preference and *η* the coefficient of inequality aversion (elasticity of marginal utility).^3^ The subscripts *i*, *j*, and *t* are the region, quintile and time indices respectively. It is clear from this specification that raising inequality aversion leads to a lower valuation of damages and mitigation costs when they fall on richer groups of individuals than if they fall on poorer groups, whether they are separated in time or contemporaneous.^4^ We solve the model by maximising the objective function (1).^5^

Most of the equations of motion are inherited from RICE. The main difference is that in NICE, regional consumption is split into five quintile shares. This is done by aggregating country income distribution data for all countries in a region (obtained from World Bank 2014) up to a regional income distribution and computing the current quintile shares for every region. We assume that within each region, pre-mitigation cost and pre-damage consumption are distributed according to this distribution and that this distribution remains constant across time.^6^

Damage and abatement costs are costs to GDP and are inherited from the RICE model. This results in impacts on regional investment and consumption. In order to attribute these regional consumption costs to sub-regional quintiles, we assume that the regional total is distributed according to constant elasticity functions with elasticities *ξ*, for damage, and *ω* for mitigation cost (see Eqs. (2) to (6) in the online supplement). For *ξ* = 1, regional damages are distributed proportionally to consumption, for *ξ* = − 1, inversely proportionally. For *ω* = 0, abatement costs fall in equal amounts on rich and poor quintiles; for *ω* = 2, they fall much more on the rich. These elasticities apply to the distribution of damage and mitigation cost *within* regions, while the total amount of regional damage and mitigation cost are determined with the regional aggregate functions from RICE.

To further illustrate the meaning of *ξ* and *ω*, consider a ‘regional economy’ comprised of two (equally populous) consumption groups A and B, with A consuming USD4000 and B USD40000 a year. If this region suffers 5% damage from climate change, they jointly lose USD2200. If *ξ* = 1, A loses 200 and B loses 2000. If *ξ* = 0, both A and B lose 1100. If *ξ* = − 1, A loses 2000 and B loses 200. B goes from losing 5 to 2.75 to 0.5%, while A goes from losing 5 to 27.5 to 50% of pre-damage consumption. If the same region experiences a 2.5% abatement cost from climate change mitigation activities, then that total joint cost to groups A and B is USD1100. If *ω* = 0, both A and B pay 550. If *ω* = 1, A pays 100 and B pays 1000. If *ω* = 2, A pays 10.9 and B pays 1089.1. A goes from paying 13.75 to 2.5 to 0.3%, and B goes from paying 1.38 to 2.5 to 2.7% of pre-mitigation cost consumption. In this way, the values of *ξ* and *ω* affect only the *distribution* of damage and mitigation cost within a region, and not the total amount of regional damage and regional mitigation cost.

The distribution of damages, and thus the value of *ξ*, depends on where and how the climate changes and modifies the ecosystem at a sub-regional level, on how vulnerable the populations are given the organisation of the economy and the infrastructure set-up, and on policy response.^7^ The value of *ξ* has not received much scrutiny so far in the empirical literature, perhaps partly due to the fact that the importance of this parameter has not previously been demonstrated. However, many studies argue that the poor will disproportionately suffer from climate change (Oppenheimer et al. 2014; Mendelsohn et al. 2006, 2011; Leichenko and O’Brien 2008; Kates 2000; Cutter et al. 2003), meaning that *ξ* is likely to be less than 1 and might even be negative (in particular in the case of health and mortality impacts). We consider that a relevant range for *ξ* in the present investigation is from − 1 to + 1.

The distribution of mitigation cost, and thus the value of *ω*, is even more dependent on policy decisions. Several studies (Bacon et al. 2010; Daioglou et al. 2012; Riahi et al. 2012; Krey 2014) analyse the share of energy in household expenditures and conclude that an increase in energy prices will hit the poor more than proportionally in the absence of compensatory measures, at least in developed nations.^8^ This suggests a value of *ω* less than 1 for a carbon tax alone with no compensatory measures. Several other studies (Cullenward et al. 2014; Metcalf 2009; Sterner 2012; Williams et al. 2014; Wilkerson et al. 2015) not only agree with the studies just cited but also conclude that if an increase in energy prices is combined with compensatory measures, it need not disproportionately hit the poor and could even make all but the highest quintile net beneficiaries—for example, if the compensatory measures involve equal per capita redistribution of the revenues from a carbon tax.^9^ In light of this, we consider that a relevant range for *ω* is from 0 to 2, the latter value being obtained when the cost is borne more heavily by the rich.

In other work (Dennig et al. 2015), we show that the value of *ξ* is of great importance to climate policy. For example, when damages are distributed inversely proportionally to income, optimal mitigation effort under the discounting and inequality aversion assumptions of Nordhaus (2007) is equivalent to optimal mitigation in the more aggregated RICE model under the much lower discounting and inequality aversion assumptions of the Stern Review (Stern 2006). At the same time, when *ξ* = *ω* = 1, inequalities within regions are fixed and are not influenced by climate change and abatement efforts. In this case, NICE produces optimal policies that are very close to those found in RICE, enabling us to obtain a good approximation of the results one would obtain with RICE in our analysis. The results of these *ξ* = *ω* = 1 model runs show that the mere representation of consumption inequalities within regions does not substantially alter the mitigation recommendations if damages and mitigation cost are proportional to consumption.

Another way in which the poor can be spared of some of the mitigation effort is by allowing different carbon prices in the different regions of the world, letting the less developed mitigate to a lesser extent than required under a uniform global carbon price (Anthoff 2009). In this paper, we do not explore this option and leave it for a companion paper in which we compare the relative importance of the within and between-region allocation of the abatement costs (Budolfson and Dennig forthcoming). As mentioned in footnote 5, we also ignore the possibility that inequality within countries could evolve in the future due to economic and policy factors and assume that it remains fixed, absent damages and abatement costs. There are reasons to believe that the distribution within countries could go either way in the future, and we leave this issue for future research.

In order to compare the quantitative importance of the distribution of income, mitigation costs and damage within regions to the importance of greater aggregate economic shocks that have been discussed prominently in the literature, we also test sensitivity to modifications of the damage functions. Following Weitzman (2012) and Dietz and Stern (2015) we consider adding a higher order temperature term (to the power of 7) to the standard quadratic damage function of the RICE model. For the ‘Weitzman’ specification, the coefficient on the higher order term is chosen so that half of GDP is wiped out by climate damages when temperatures reach 6 °C above pre-industrial levels. For the ‘Dietz-Stern’ specification, the coefficient is chosen so that half of GDP is already wiped out at a temperature increase of 4 °C above pre-industrial. For comparison, for the median region, the RICE damages yield approximately 4 and 8% GDP losses at 4 and 6 °C respectively.^10^

As in RICE, there is no uncertainty in our model. Instead, we consider the possibility of such strong convexities in damage *with certainty* and analyse the sensitivity of the optimal carbon price to the different specifications.

## Results

### The distribution of sub-regional costs and benefits, and the Schelling Reversal

The main innovation in NICE is the more detailed description of intragenerational inequality and the possible ways in which damages and mitigation costs may affect it. Notice that the specification (1), via the inequality aversion parameter *η*, picks up on inequality in a similar way, whether it is across time or across space.

Here, we find that whether the optimal carbon price increases or decreases with inequality aversion depends on whether the intergenerational or intragenerational inequalities dominate the evaluation. The balance depends on a variety of factors, which we illustrate by plotting several panels with optimal prices for values of the elasticity *η* ∈ {0, 1, 2, 3} within each panel.

**Fig. 1:**
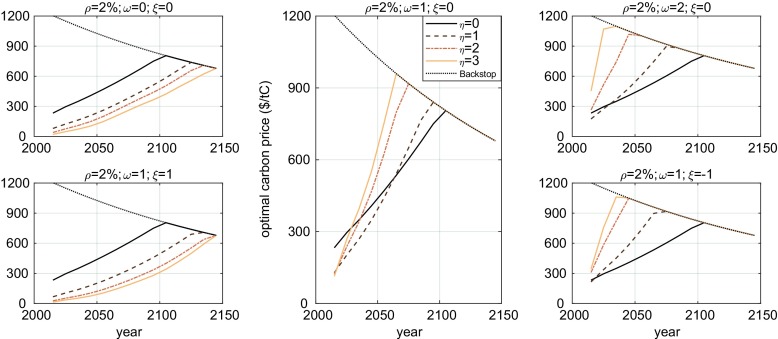
Optimal carbon price paths for different values of *η* under five different assumptions for the distribution of mitigation costs and climate damage within regions, all with *ρ* = 2% and the RICE damage functions. The central assumption (of the middle panel) is that mitigation cost is distributed proportionally to income and climate damage is independent of income (*ω* = 1 and *ξ* = 0). Around that assumption, we consider a greater and a lower income elasticity of mitigation cost (first row) and a lower and greater income elasticity of damage (second row). Note two things: first, over the chosen parameter ranges, decreasing the mitigation elasticity and increasing the damage elasticity by ± 1 have a quantitatively similar effect, which is quantitatively important for all values of *η* ≥ 1. Second, in the leftmost column, increasing inequality aversion leads to lower optimal mitigation of the implication of the Ramsey equation, while the middle and rightmost column exhibit a reversal of that relationship. The line corresponding to *η* = 0 is not just similar, but theoretically identical across panels. This assists the comparison across panels

In Fig. 1, we plot five such panels for different values of the mitigation cost and damage elasticities of income and a rate of pure time preference of *ρ*=2%. The figure also plots the backstop price, which all price paths eventually join at the date of full mitigation. This is the carbon price at which zero emission technologies are assumed to be competitive with fossil fuels, thereby displacing their use completely.

At zero inequality aversion (*η* = 0), the optimal price is the same across panels and high ($233/tC in 2015) relative to commonly computed values with IAMs (e.g. Nordhaus 2010). It is high because future damages are not discounted relative to current costs on account of future generations being richer. It is the same across panels because with such social preferences, the distribution of costs and benefits does not matter, so the values taken by *ξ* and *ω* are irrelevant to the optimal price. In Dennig et al. (2015), we showed that when *η* = 2, different assumptions about the distribution of damage resulted in very different optimal carbon prices. Here, we establish (by comparing lines of the same colour across panels) that this is true for a range of values of *η* and equally true for the distribution of mitigation cost. The greater *η*, the more important it is to get the correct value of the elasticity parameters *ξ* and *ω*.^11^

Increasing inequality aversion results in lower optimal prices in the two panels in the left column. These are the circumstances under which the current poor are more prominent in the evaluation, and thus, the familiar logic of the Ramsey equation applies. This is when the distribution of mitigation costs is more regressive (top row) or when the distribution of damage is more progressive (bottom row). In the first case, less mitigation strongly benefits the current generations’ poorest, so more concern for the poor reduces optimal mitigation. In the second case, more mitigation benefits the future rich more than the poor, so more inequality aversion again favours the current poor inducing less mitigation.

The opposite is true in the rightmost column. Here, the future rather than the present poor dominate the evaluation, and the Ramsey equation is upended, as Schelling conjectured might happen. In the top row, mitigation costs are borne largely by the current rich. This shields the current poor from increases in mitigation, making greater inequality aversion result in higher optimal prices.^12^ In the bottom row, future damages are borne predominantly by the poor rather than the rich. Greater inequality aversion therefore increases optimal carbon prices to protect them, even if on aggregate the future is much richer. This verifies Schelling’s conjecture that a reversal of the effect of inequality aversion is possible once sub-regional inequalities are properly represented.

Under the central assumption in the middle panel, the balance favours greater carbon prices at higher inequality aversion in the future, but an opposite (small) effect for present prices. This highlights that there is also a dynamic component to the interaction between the distribution of costs and benefits and inequality aversion. Relative to the left column, the price paths become steeper with greater inequality aversion. Under those assumptions, greater inequality aversion results in a present reduction in mitigation effort because it improves the situation of the current poor. However, as economic growth increases the living standard of the poorest in the near future, the optimal price increases rapidly if inequality aversion is high. In the supplemental material, we produce companion figures to Fig. 1 showing the same panels for lower rates of time preference. As is well documented in the literature, lower discount rates imply higher optimal prices (see Nordhaus 2007; Weitzman 2007; Dasgupta 2008). In many cases, time preference does not interact with inequality. But for *ρ* = 0%, for example, we find that the Schelling Reversal does not take place: all panels have optimal prices that decrease with *η*. This is because of a level effect. At such a low discount rate, the optimal prices are already so high that the most important damages are avoided. The optimum is already skewed in favour of the future, so increasing inequality aversion is more sensitive to the present poor, irrespective of the distribution of costs and damages. When *ρ* = 1%, we have an intermediate case where the effects balance out and the optimal price is not particularly sensitive to *η*.

Altogether, these results reveal the supreme importance of the distribution in income, damages and mitigation costs in the determination of the optimal carbon price. They confirm that Schelling’s Reversal can occur and that this depends in an intuitive way on the distribution of damages and mitigation costs within regions: the more the poor stand to benefit from mitigation and the less they pay for it, the more likely the reversal. However, this also depends on the rate of pure time preference, which must be sufficiently high (and imply a sufficiently modest mitigation) for the reversal to be observed.

### Interactions with catastrophic damages

So far, we have taken the total dollar amount of economic damage resulting from climate change as given by the original quadratic damage specification of the RICE model, and only modulated (by changing *ξ*) the way in which that total amount of damage is distributed across the income groups of every region. Here, we consider two additional damage specifications in which there is significantly greater overall damage once temperatures increase beyond what has been observed in the historic record. As described at the end of Sect. 2, we have two additional specifications: ‘Weitzman’ and ‘Dietz-Stern’.

The direction of the effect on optimal prices of greater damages is obvious: the specifications with greater damage lead to greater optimal mitigation. However, surprisingly, for most reasonable values taken by the other parameters, the effect of adding such huge damages *with certainty* has a comparatively small effect in the optima when compared with the effect of the distributional parameters investigated here.

**Fig. 2:**
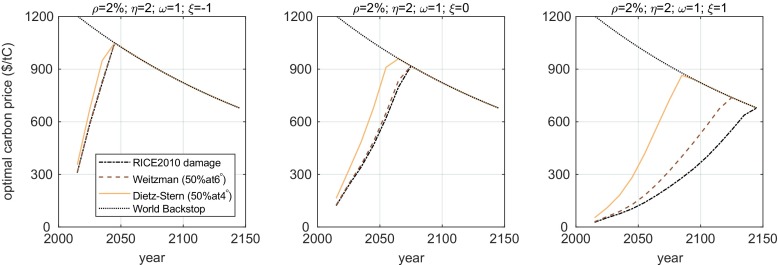
Optimal carbon price paths for different damage function specifications under the three different values for the income elasticity of damage, *ξ*, all with *ρ* = 2%, *η* = 2 and *ω* = 1. Notice that as you move to the right across panels, the greater value of *ξ* results in lower prices, and it is only when the prices are sufficiently low that the optimum across damage specifications is significantly different. Varying *ξ* under RICE damages (dashed-dotted lines across panels) has a greater effect than varying the damage specification (comparing lines within a panel)

In Fig. 2, we plot optimal prices for different assumptions about the total damage and different income elasticities of damage *ξ* (for *ρ* = 2 % , *η* = 2, and *ω* = 1). As described at the end of Sect. 2, the damage specifications are calibrated to entail 50% damage to global output at a 6 °C (as in Weitzman 2012), or at 4 °C (Dietz and Stern 2015). (These assumptions are described in more detail in the online appendix).

In the left panel, the elasticity of damage is low, and therefore, the optimal carbon prices are high. Comparing the different damage specifications, we see that all three are almost identical. If, however, the elasticity of damage is high (right panel), the optimal prices and mitigation efforts are smaller, and the difference across damage specifications becomes more significant.

**Fig. 3:**
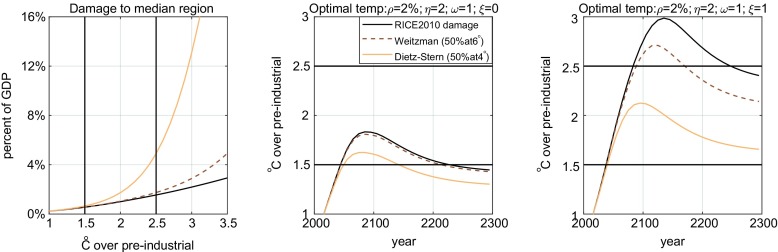
The left panel plots the damage function of the median region, which we define as the average of the damages of the regions with the sixth and seventh largest damages (the model has 12 regions). The corresponding regions are ‘Europe’ and ‘other non-OECD Asia’. The middle and right panels plot the atmospheric temperature paths at the optima described in the corresponding panels of Fig. 2. Notice that in the middle panel, the RICE optimum and the Weitzman optimum are below 2.5 °C. Below that temperature, the RICE and Weitzman damages are barely distinguishable, which is why the two optima are so similar. The Dietz-Stern damage is significantly different from the other two beyond 1.5 °C: a temperature surpassed by the other optima in the middle panel

The results in Fig. 2 are best explained by looking at the temperatures reached at the different optima and at how different the damage specifications are at those temperatures. As can be seen from the leftmost panel of Fig. 3, there is hardly a difference between the three damage functions below 1.5 °C. Any mitigation path that avoids higher temperatures even for the RICE 2010 damages will not change perceptively when the higher order damage term is introduced. This is the case for the carbon prices in the left panel of Fig. 2. The difference between Weitzman (50% at 6 °C) and RICE 2010 only becomes noticeable past 2.5 °C. So, if the RICE damage optimum results in maximum temperatures between 1. 5 and 2.5 °C, the Weitzman damage optimum will not differ significantly from it, but the Dietz-Stern damage optimum will. This is illustrated in the middle panel of Fig. 3, which corresponds to the optimal prices in the middle panel of Fig. 2. Finally, if the RICE damage optimum results in maximum temperatures above 2.5 °C, then the Weitzman damage optimum will differ significantly from it, as illustrated in the right panels of Figs. 2 and 3.

We note that while this analysis holds for optimal carbon prices, the economic damage that results from having a non-optimal policy (i.e. a policy that is too lax) may be quite large and significantly affected by the alternative assumptions about damage considered here. Incorporating uncertainty over the damage function and other key parameters (including climate sensitivity and other parameters not even discussed here) might also lead to a stronger policy response.

In the supplemental material, we have included plots corresponding to the three panels of Fig. 2 for different values of *ρ*, *η*, and *ω*. The overall pattern is similar to the one in Fig. 2. The different damage specifications matter more when the effort under the RICE specification is low (i.e. for high values of *ρ* and low values of *ω*). The effect of *η* cannot be summarised in such a way because of the ambiguous effect of *η* on the optimal policy.

Weitzman (2007) disagreed strongly with the authors of the Stern Review regarding their choice of normative parameters (*ρ* and *η*), but agreed to some extent that high levels of effort are warranted if uncertainty is taken into account, with a low probability of very strong damages on aggregate output. Qualitatively, this effect can be seen in the right panel of Fig. 2. The normative parameters and damage *distribution* assumptions are similar to what Weitzman had in mind, and there is a clear effect.

However, when optimal mitigation effort is already high, as is the case with a highly regressive damage distribution, the inclusion of an aggregate catastrophe in the damage function has no effect on the optimum. Furthermore, comparing the variation in optimal carbon prices for the range of values taken by the distributional parameters (the income elasticities *ξ* and *ω*) with the variation due to the different aggregate damage specifications, it becomes apparent that the way in which costs and benefits are distributed across income groups has a much greater impact on the optimal carbon price than even the extreme damage specification by Dietz-Stern. Only if *η* = 0 do distributional considerations become less important that the possible aggregate catastrophe, and no example in the IAM literature considers that possibility. This is an important lesson. By neglecting the intragenerational distribution of inequalities (within regions and countries), the literature had to look at global catastrophes in order to explore potential reasons for strong mitigation. With inequalities and a skewed damage distribution, the social catastrophe looms large even at less extreme temperature increases.

## Conclusion

NICE reveals the importance of a factor largely ignored by existing IAMs: the interaction between climate change and inequalities within regions and countries. But disaggregating the regions of the world into income strata does not by itself suffice to overturn the usual conclusions. A greater inequality aversion in the social welfare objective pursued by policy may enhance or undermine the recommended level of mitigation depending on the other parameters and assumptions. The evaluation of the Schelling Reversal therefore depends in various (sometimes obvious, sometimes subtle) ways on several conditions. As Schelling argued, the distribution of damages and abatement costs is the essential ingredient in the analysis and involves both climate, economic and political assumptions.

Using NICE, we have investigated the interactions between a number of factors that can, in isolation, have an important effect on the strength of optimal mitigation—namely, pure time preference, inequality aversion, inequalities in the distribution of both damage and mitigation cost between rich and poor and a damage function that does or does not assume catastrophic impacts at high global mean temperatures increases. We have shown the comparative importance of these factors by displaying optimal carbon price trajectories that arise from the wide variety of combinations that are possible given the primary range of disagreement over each factor.

To frame the discussion, we began by articulating Schelling’s conjecture that properly accounting for inequalities could lead the inequality aversion parameter to have an effect opposite to what is suggested by the Ramsey equation. We showed that the Schelling Reversal does indeed happen but only when a set of conditions are all satisfied: roughly, when climate damages disproportionately harm the poor, when mitigation costs are not disproportionately paid by the poor, and when discounting is sufficiently high making effort at the optimum low; in these circumstances, increasing inequality aversion leads to faster optimal mitigation. Otherwise, intergenerational inequalities tend to dominate. The Schelling Reversal emerges especially strongly in situations of a progressive distribution of abatement costs and a regressive distribution of climate damages.

We also showed that for most values taken by the other parameters, the effect on optimal policy of adding catastrophic damages with certainty at 4 or 6 °C above pre-industrial temperatures typically has a smaller effect than the other parameters investigated here, especially when compared to the effect of sub-regional inequalities. The addition of potentially catastrophic damages has a larger effect on optimal policy when damages are proportional to income than when they fall more on the poor, because the latter requires strong mitigation for reasons independent of the potential for catastrophic damages, which prevents temperature from increasing to the point where the elevated damage function would be relevant. However, we note that our experiments are deterministic and do not explore uncertainty as well as the economic consequences of non-optimal carbon policies, both of which are important further issues, and which we explore in work in progress.

In sum, NICE provides answers to a number of pressing questions about the comparative importance of familiar factors such as discounting and higher order damage terms for the determination of the price of carbon. This paper has especially highlighted that distributional catastrophes may loom larger than global catastrophes, although inequality aversion tends to shift the focus towards the future poor rather than the present poor only when time preference is high, i.e. when mitigation is low.

## Electronic supplementary material

ESM 1 (PDF 1285 kb)

## Footnotes

1 A related conjecture from Schelling is investigated by Anthoff and Tol (2012)—namely, that the best defence for poor societies against climate change impacts might be to develop quickly, rather than to control greenhouse gas emissions. Our results here shed additional light on this and other important questions framed and partially answered by Anthoff and Tol. Our results here indicate that proper representation of subregional inequalities is crucial to answering such questions—which supports Anthoff and Tol’s idea that answering such questions requires taking into account important heterogeneities (p. 271) and echoes Anthoff et al.’s (2009) plea to introduce intra-national inequalities in the future research.

2 It is in particular noteworthy that Stern had to choose a low degree of inequality aversion in order to obtain a low discount rate and to recommend an aggressive mitigation policy, whereas Nordhaus adopted a more standard inequality aversion parameter (and higher discount rate) and obtained much more lenient conclusions. On the use of discount rates in policy, see the recent synthesis in Groom and Hepburn (2017).

3 Ignoring the discount rate, the social objective (1) considers that a population at half the consumption level of another should receive a greater priority by a factor of 2^*η*^ for small increments to consumption (e.g. a value of *η* = 1 means that providing $1 to the poorer population is as good as providing $2 to the less poor population).

4 In this model, environmental services are not separated from consumption. Hoel and Sterner (2007) and Sterner and Persson (2008) show that distinguishing the two types of goods may make quite a difference, if the elasticity of substitution between consumption and environmental services is low. We leave this issue for future research.

5 The maximisation of this objective results in the welfare optimal, rather than non-cooperative solution. Furthermore, we do not use Negishi weights.

6 We have not considered changes in this distribution over time. Qualitatively, it is clear that if this distribution equalises over time, our results become more similar to those of the RICE model, while if the distribution becomes less equal over time, the divergence between our results and the RICE model becomes more important. We leave the quantitative analysis of such scenarios to future work.

7 Note that the measurement of damages itself has both empirical and ethical dimensions: valuing losses to different parts of the income distribution in the wake of climate change depends both on relatively objective data on property damage, capital losses, etc., and the more ethically challenging questions regarding valuation of loss of life, health and livelihood.

8 The papers in Sterner (2012) suggest that even without compensatory measures, a carbon tax in developing nations might not be regressive.

9 As a consequence, progressive compensatory measures can also arguably improve the political feasibility of carbon taxes, at least as measured by percentage of voters who are net beneficiaries of the policy.

10 The damage of the median region is computed as the mean of the damages of the regions with the sixth and seventh greatest damages (out of 12 regions). At 4°, these are ‘OECD Europe’ and ‘other non-OECD Asia’, and at 6°, these are ‘Middle East’ and ‘other non-OECD Asia’. The region with the maximum damage is ‘China’ at 4° with 7% and ‘China’ at 6° with 14%.

11 Note that for some values of these elasticities, increasing *η* results in lower carbon prices than the price at *η* = 0 (which is the same across panels), and for other values, increasing *η* results in greater prices.

12 If the mitigation costs were distributed in a way that made all but the richest quintile net beneficiaries (Metcalf 2009; Sterner 2012; Wilkerson et al. 2015; Williams et al. 2014), the results would presumably be even starker.
