# Supplementary material for: The comparative importance for optimal climate policy of discounting, inequalities and catastrophes
Source: Clim Change. 2017 Oct 28;145(3):481–94. doi: 10.1007/s10584-017-2094-x (PMC6956944; doi:10.1007/s10584-017-2094-x)
Supplement: Supplementary file 1 — (PDF 1285 kb) [file 10584_2017_2094_MOESM1_ESM.pdf]

Online Supplement

In this supplement we describe the NICE Model used for this analysis and present sensitivity analyses to the results shown in the main article.

1. The NICE Model

In this section we describe the NICE model that is used in our analysis, which evaluates public policy with a social welfare function. Following RICE2010 (Nordhaus 2010), on which NICE is based, and most of the literature, we use a discounted and separable constant elasticity function with population weights:

$$W\left(c_{ijt}\right) = \sum_{ijt} \frac{L_{ijt}}{(1+\rho)^t} \frac{c_{ijt}^{1-\eta}}{1-\eta} \tag{1}$$

where W denotes social welfare, L population, c per capita consumption, ρ the rate of pure time preference and η inequality aversion. The subscripts *i, j*, and *t* are the region, quintile, and time indices respectively. RICE only has region and time indices, while the quintiles are novel to NICE.

In NICE and RICE the world is composed of twelve macro region economies 0 (some of which are in fact countries, like China, India, the USA, Russia, and Japan). The basic policy trade-off is between output today – through greater or smaller mitigation cost – and output tomorrow – through greater or lower climate damages. As in RICE, *net regional output* in period *t* is given by

$$Y_{it} = \left(\frac{1-\Lambda_{it}}{1+D_{it}}\right) Q_{it} \tag{2}$$

where Q_{it} is gross output, D_{it} is regional damage and Λ_{it} is regional mitigation cost.

For regional population L_{it} and savings rate s_{it}, investment is

$$I_{it} = s_{it} Y_{it} \tag{3}$$

and average per capita consumption (for region *i* at time *t*) is

$$\bar{c}_{it} = \frac{1-s_{it}}{L_{it}} Y_{it} \tag{4}$$

Up to this point, we have described the RICE model, which coincides with NICE on those points.[1] In order to investigate the ways in which climate interacts with a more fine-grained representation of inequality, we further refine the regional consumption in equation (4) to represent the sub-regional consumption distribution. As an approximation of the regional consumption distribution we compute the quintile share, q_{ij} of quintile *j* in region *i* by aggregating the country income distribution data from the World Bank Development Indicators (World Bank 2014). We assume that these distributions are a proxy

[1] RICE and NICE coincide on equations (2) – (4). The social objective is different in that NICE uses (1) and RICE uses a Negishi weighted social objective.

for consumption distributions before accounting for climate damages and mitigation cost, and remain constant into the future.[2] On that basis we compute pre-damage and pre-mitigation cost per capita consumption level per quintile from each region's per capita consumption in each period. (Note here and in the equations that follow that this leaves regional climate and economy aggregates as computed in RICE2010.)

From here, the first novel equation of NICE computes the pre-mitigation cost and pre-damage per capita consumption of quintile j in region i as

$$c_{ijt}^{pre} = \bar{c}_{it}\left(\frac{1+D_{it}}{1-\Lambda_{it}}\right)q_{ij} \quad (5)$$

where q_{ij} is the consumption share of quintile j in region i. In analogy to the consumption shares we define the mitigation cost and damage shares of quintile j in region i by e_{ij} and d_{ij} respectively.[3]

Notice that because total abatement cost is deducted first (in the numerator) and damage cost is deduce second (in the denominator) in equation (2), the total abatement cost to per-capita consumption in region i is $\bar{c}_{it}\left(\frac{1+D_{it}}{1-\Lambda_{it}}\right)\Lambda_{it}$ while the total damage cost to per-capita consumption in region i is $\bar{c}_{it}D_{it}$.[4] Consequently, the mitigation cost of quintile j in region i is $\bar{c}_{it}\left(\frac{1+D_{it}}{1-\Lambda_{it}}\right)\Lambda_{it}e_{ij}$ and the damage cost to the same group is $\bar{c}_{it}D_{it}d_{ij}$.

Therefore post-damage and post-mitigation cost average per capita consumption (for quintile j in region i at time t) is given by by

$$c_{ijt} = c_{ijt}^{pre} - \bar{c}_{it}\left(\frac{1+D_{it}}{1-\Lambda_{it}}\right)\Lambda_{it}e_{ij} - \bar{c}_{ij}D_{it}d_{ij} \quad (6)$$

In order to consider different mitigation cost and damage distributions by varying a simple parameter we assume a constant elasticity relationship within regions between the mitigation cost and the consumption shares, as well as between the damage and consumption shares. Denoting the elasticity parameters by ω and ξ the mitigation cost and damage shares are given by[5]

---

[2] The assumption that they remain constant is motivated by the fact that, whereas one can hope for the inequalities across regions to decrease in the future (as assumed in RICE and reproduced in NICE) as a result of economic and technological convergence, the inequalities within countries are submitted to opposing forces which make it possible to observe changes in either direction.

[3] As defined here q_{ij}, e_{ij} and d_{ij} are actually the quintile shares multiplied by 5. We do this for ease of exposition, so that we don't have to carry a factor of 5 in all computations of per capita values. For example, if the consumption distribution were uniform the actual quintile shares would be $q_{ij} = 1/5$ for all quintiles j. But instead we set $q_{ij} = 1$, so that equations (5) and (6) correctly attribute the regional per capita value to each quintile.

[4] The total costs to GDP (both damage and abatement) equal the cost to per-capital consumption times the population, plus the cost to investment, which we leave unaltered relative to RICE.

[5] Recall that the quintile shares are fixed and computed by aggregating country level distributional data to the regional level.

$$e_{ij} = k_{i\omega} q_{ij}^{\omega}; \quad d_{ij} = k_{i\xi} q_{ij}^{\xi}. ; \qquad (7)^{6}$$

By modifying the parameters ξ and ω, we are thus able to vary the distribution between quintiles of mitigation costs and climate damages. For ξ = 1, regional damages are distributed proportional to consumption; for ξ = -1, inversely proportional. For ω = 0, abatement costs fall in equal amounts on rich and poor quintiles; for ω = 2, they fall much more on the rich.

Recent literature has suggested that the economic consequences of climate change may be more pronounced than previously thought, and become larger and more uncertain at greater departures from preindustrial temperatures. This has motivated some economists to argue for the importance of using a "fat-tail" approach to modelling the risk of low probability but very high impact scenarios that may emerge from an altered climate (Weitzman 2011, 2013). (See Houser et al. 2014 and Burke et al. 2015 for empirical arguments that damages may be larger than previous estimates.)

In RICE2010, the function determining the damage term D_{it} is a quadratic function of temperature rise above preindustrial levels (T):

$$D_{it} = \alpha_{1i} T_t + \alpha_{2i} T_t^2$$

Its coefficients are based on the empirical estimates described in the introduction, which have a domain of approximate validity near and below 2.5°C (Tol 2009, Nordhaus 2013). In an effort to model the possibility of extreme damage beyond that domain, but lacking much empirical basis for extrapolation, Weitzman (2012) proposed adding a higher order temperature term based on a thought experiment of what could happen at a temperature of 6°C. To this end, in NICE, we add an additional term to the RICE2010 damage function that applies a coefficient to temperature raised to the 7th power, in order to explicitly consider the possibility that climate change could have a much more catastrophic impact on the global economy:

$$D_{it} = \alpha_{1i} T_t + \alpha_{2i} T_t^2 + \alpha_U T_t^7 \qquad (8)^{7}$$

Weitzman 2012 supposes that damages equal to 50% of gross output would occur at T = 6°C. In a recent paper focusing on damages on capital, Dietz and Stern (2015) imagine an even gloomier calibration with 50% damage at T = 4°C. We consider optimal taxes for all three of these specifications: original RICE2010, Weitzman, and Dietz-Stern, and calibrate the α_U parameter accordingly. (For the Weitzman case α_U= 3.3615e-06. For the Dietz-Stern case α_U= 5.8707e-05.)

2. Capital accumulation and the savings rate

[6] For equation 7, the parameter values $k_{i\xi}$ and $k_{i\omega}$ are chosen such that $\sum_j d_{ij} = 1$ and $\sum_j e_{ij} = 1$ respectively.

[7] In the RICE model, the damage coefficients on the linear and quadratic terms are region specific. In our initial exploration here, this added α_U term is not regionally specific but rather indicates damage to the global economy as a result of climate change.

The calibration for the RICE model yields annual rates for several variables, which are then cumulated to the decadal time step of the model. In particular, in the RICE2010 spreadsheet the assumed capital depreciation rate of 10% per annum is cumulated geometrically to the 10 year time step, while savings are accumulated arithmetically. Specifically, capital is accumulated over the 10 year time step as:

$$K_{t+10} = K_t * (1 - \delta)^{10} + 10 * I_t \qquad (9)$$

where I_t is annual investment. If one puts faith in the calibration of annual rates, this combination of geometric depreciation and arithmetic accumulation quite significantly overstates capital accumulation at the ten year step, while combining geometric accumulation and depreciation or arithmetic accumulation and depreciation is more accurate.[8] In our version of the model we have made both accumulation as well as depreciation arithmetic, yielding

$$K_{t+10} = K_t * (1 - 10\delta) + \ 10 * I_t \qquad (10)$$

which is closer to the correct accumulation over the 10 year time step.

We also modify the savings rates. In RICE2010 the regional savings rates are chosen to maximise the Negishi weighted global welfare function, taking optimal carbon taxes as given. This is computationally cumbersome, and unlikely to be a reasonable description of savings rates, as it overstates the savings rates of regions with high growth rates and understates savings rates of regions with low growth rates relative to what representative agents in such regions would choose.[9] The savings rates of the RICE model are reproduced in Table 1 below. Instead, we apply a fixed Solow savings rate of 25.8% in every time period and every region. This vastly simplifies the modelling, and could even be considered an improvement over the savings rates in the original model, since it can be rationalised as the optimal savings rate as determined by an infinitely lived agent with logarithmic utility and a utility discount rate of 1.5% per annum.[10] While the optimal rates according to a global Negishi weighted objective cannot be rationalised by any regional representative agent.

The net effect of the two changes to savings and capital accumulation is that there is slightly less capital accumulation in our version than in RICE2010. In fact, if our model had a fixed rate of 30% the capital accumulation in our version would be approximately equal to that in RICE2010 for most of the model horizon. Having the lower savings rate of 25.8% affects the values of aggregate variables such as GDP and emissions at the optimum, but it has only a small effect on the value of the optimal carbon tax, as can be seen in Figure SM1 below. Recall that the 25.8% is arrived at by assuming logarithmic utility and a utility

---

[8] To see this notice that the correct first order approximation is

$$K_{t+10} = K_t(1 - \delta)^{10} + I_t \sum_{j=0 \ to \ 9} (1 - \delta)^j$$

When $\delta = 0$ all three variants are the yield the same capital ten periods later. But if δ becomes large enough for the linear approximation of the logarithm to become inaccurate, then the version used in RICE2010 diverges significantly from the correct accumulation.

[9] See Dennig & Emmerling (2017) for this result.

[10] See Golosov et al (2014) for this result.

discount rate of 1.5% per annum along with complete depreciation. If one were to assume an elasticity of marginal utility of, say 1.5 or 2, the savings rate would be even lower, making capital accumulation diverge even more from the RICE model.

The preceding sections summarized the key features of NICE relevant to the analysis in this paper. Additional technical discussion of NICE and its relation to RICE2010 is available in the methods, appendix, and online supplement of Dennig et al. 2015.

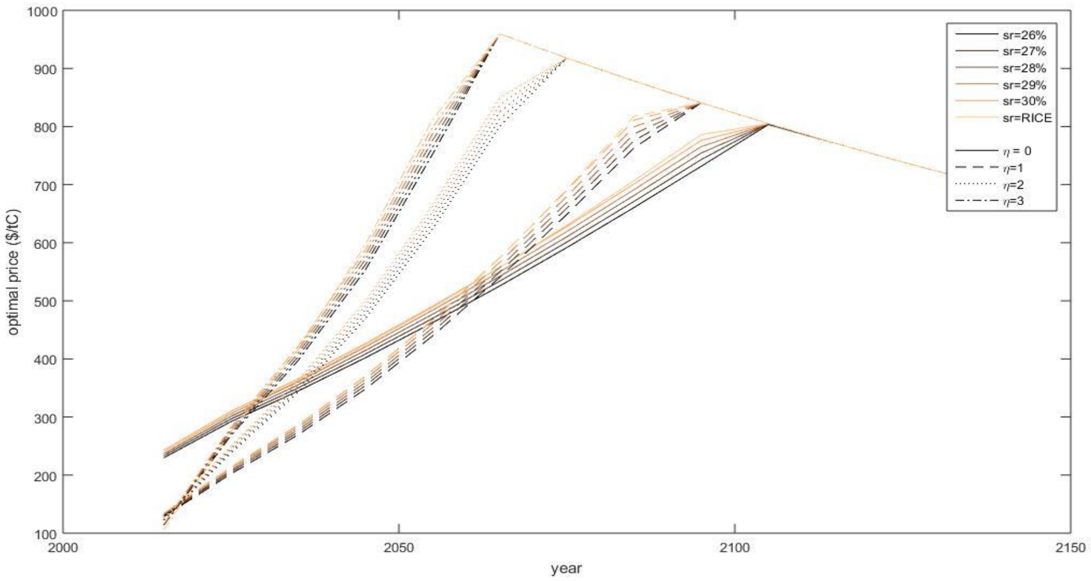

Figure SM1: Four groups of optimal carbon price paths for 6 different assumptions on capital accumulation. Five assumptions correspond to fixed savings rates (of different magnitudes) along with full capital depreciation over a decade. The sixth corresponds to the exact savings rates and capital depreciation assumptions of the original RICE2010 spreadsheet. The different groups correspond to different values of the elasticity of marginal utility, η. The other parameters correspond to the values assumed in the middle panel of Figure 1 in the main text ($\rho = 2\%, \xi = 0, \omega = 1$).

year	2005	2015	2025	2035	2045	2055	2065	2075	2085	2095	2105	2115
US	0.18	0.20	0.20	0.20	0.20	0.20	0.20	0.20	0.21	0.21	0.21	0.22
EU	0.17	0.20	0.20	0.19	0.19	0.19	0.19	0.20	0.20	0.21	0.21	0.22
Japan	0.15	0.18	0.18	0.17	0.18	0.19	0.19	0.19	0.19	0.20	0.21	0.22
Russia	0.19	0.19	0.19	0.19	0.19	0.19	0.19	0.19	0.19	0.20	0.21	0.22
EurAs	0.20	0.23	0.22	0.21	0.21	0.21	0.21	0.21	0.21	0.22	0.22	0.23
China	0.36	0.23	0.22	0.21	0.20	0.20	0.20	0.20	0.20	0.21	0.22	0.21
India	0.29	0.27	0.25	0.24	0.23	0.23	0.23	0.22	0.22	0.22	0.23	0.23
MidEst	0.26	0.25	0.24	0.23	0.22	0.22	0.22	0.22	0.22	0.22	0.21	0.22
Africa	0.30	0.30	0.29	0.28	0.26	0.24	0.24	0.24	0.24	0.23	0.22	0.22
LatAm	0.24	0.24	0.23	0.22	0.21	0.21	0.21	0.21	0.21	0.22	0.22	0.23
OHI	0.19	0.20	0.20	0.19	0.19	0.19	0.19	0.19	0.19	0.20	0.21	0.22
Other	0.24	0.28	0.27	0.25	0.24	0.23	0.23	0.23	0.23	0.23	0.23	0.22

Table 1: Savings rates in Nordhaus's RICE2010 optimum. Notice that these are combined with less capital depreciation (0.9^{10} over a decadal time step, compared with full depreciation in our version), resulting in significantly greater capital accumulation than in our model runs with a 25.8% savings rate. These savings rates are roughly equivalent to a 30% savings rate in a model with full capital depreciation over a decade.

3. Backstop prices

The backstop price is the carbon price at which full abatement takes place. The idea is that at such a high penalty for emissions, non-fossil sources of energy become competitive. This is a standard concept in the theory of exhaustible resources (Dasgupta and Heal 1979). The RICE model posits a global backstop price that declines over time, and attributes to each region a fraction (or multiple) of that price. The US, for instance, has a backstop of 0.9 times the world backstop, and India has a backstop of 1.1 times the world backstop. For the runs in this paper we have set the backstop prices of all regions to equal the world backstop price. This is done because having different backstops lead to non-differentiabilities in the objective as the carbon price passes from below the backstop of a region to above the backstop. This leads to kinks in the optimal carbon price that obscure the sensitivity to parameters for paths that are very similar. As can be seen from Figure SM2, the path taken by the optimal carbon prices up until the global backstop is very similar, regardless of what assumption was made about the backstop.

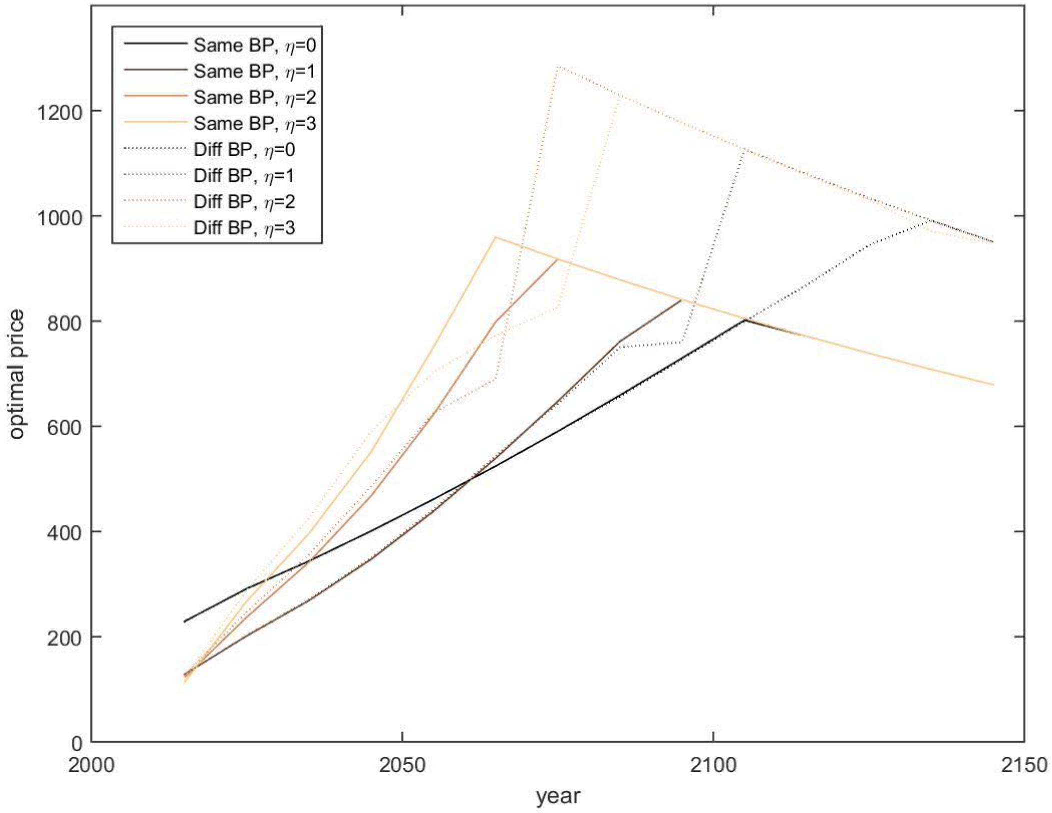

Figure SM2: Optimal carbon price paths corresponding to the optima in the middle panel of Figure 1 in the main text, but for two different assumptions about the backstop prices. The solid lines assume that all the regions have a backstop price equal to the worlds backstop price, while the dotted lines assume that each region has a different backstop, as assumed in the original RICE model. The four different optimal being replicated correspond to different values of the elasticity of marginal utility, η. The other parameters correspond the ones in the middle panel of Figure 1 in the main text $(\rho = 2\%, \xi = 0, \omega = 1)$

4. Additional results referred to in the main text

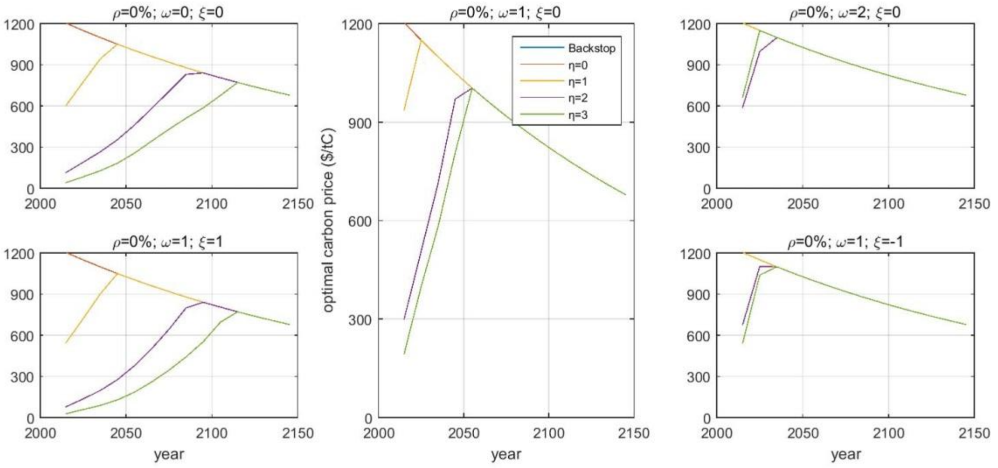

Figure 1A: Optimal carbon price paths for different values of η under five different assumptions for the distribution of mitigation costs and climate damage within regions, all with $\rho = 0\%$ and the RICE damage functions. Similar to Figure 1 in main text, but with a different value of ρ.

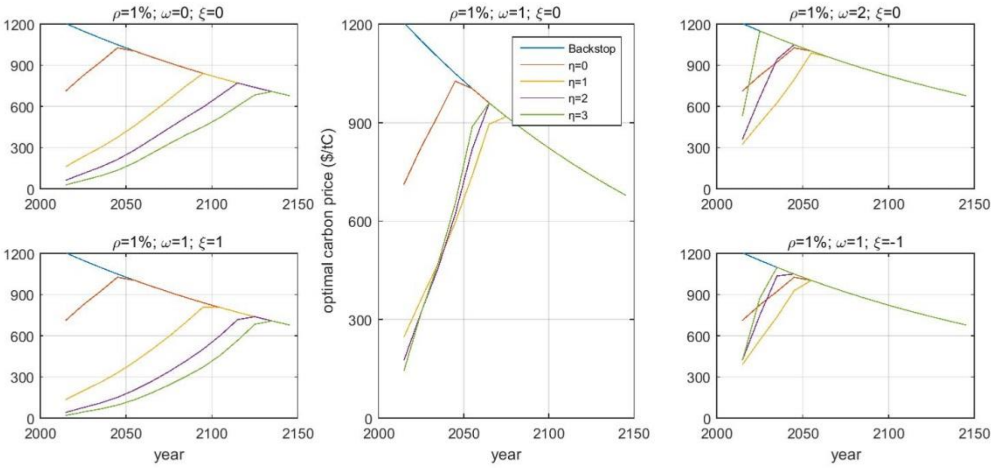

Figure 1B: Optimal carbon price paths for different values of η under five different assumptions for the distribution of mitigation costs and climate damage within regions, all with $\rho = 1\%$ and the RICE damage functions. Similar to Figure 1 in main text, but with a different value of ρ.

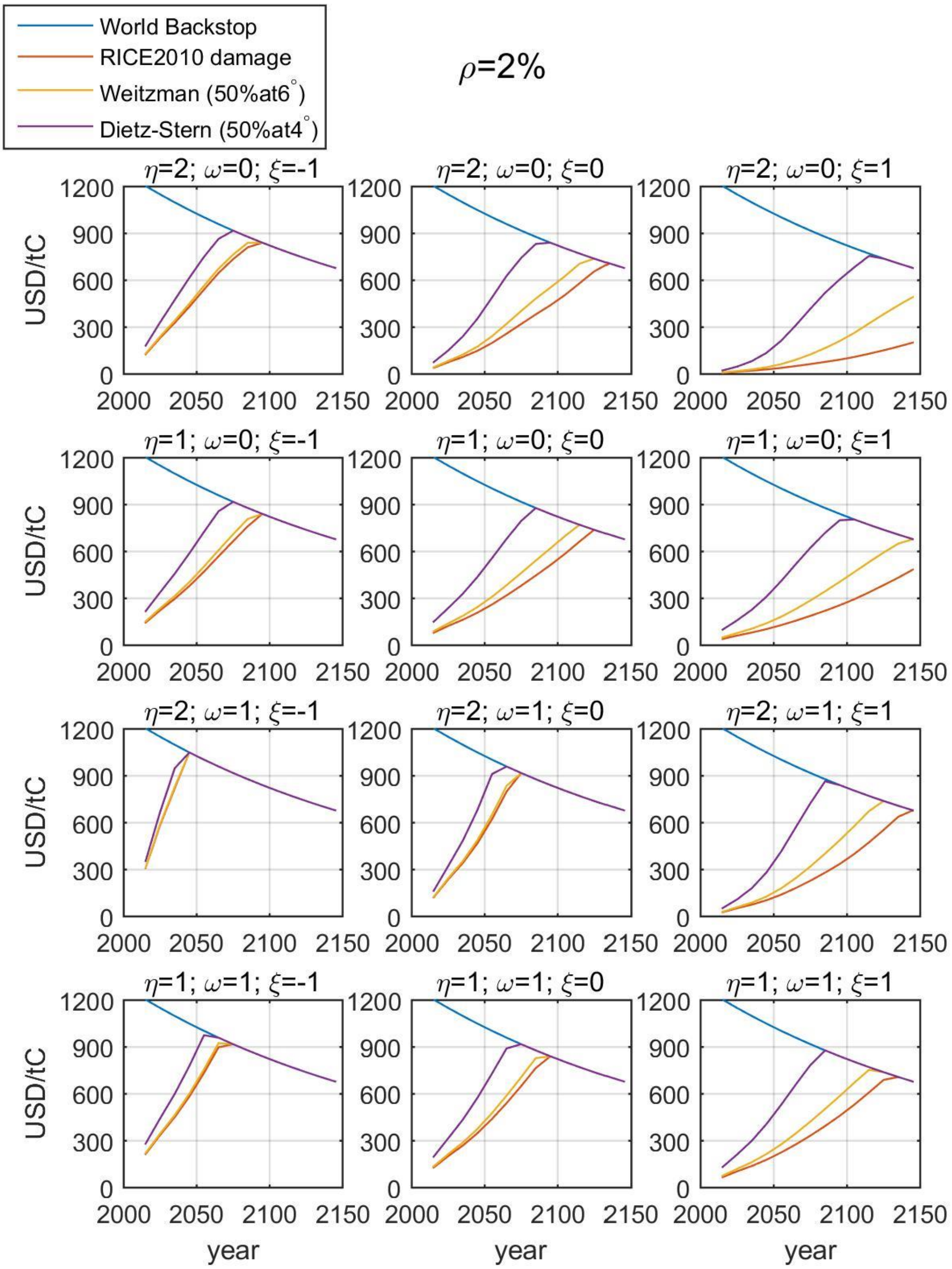

Figure 2A: Optimal carbon price paths for different damage function specifications under three different values for the income elasticity of damage, ξ, for combinations of $\eta \in \{1, 2\}$ and $\omega \in \{0,1\}$, all with $\rho = 2\%$.

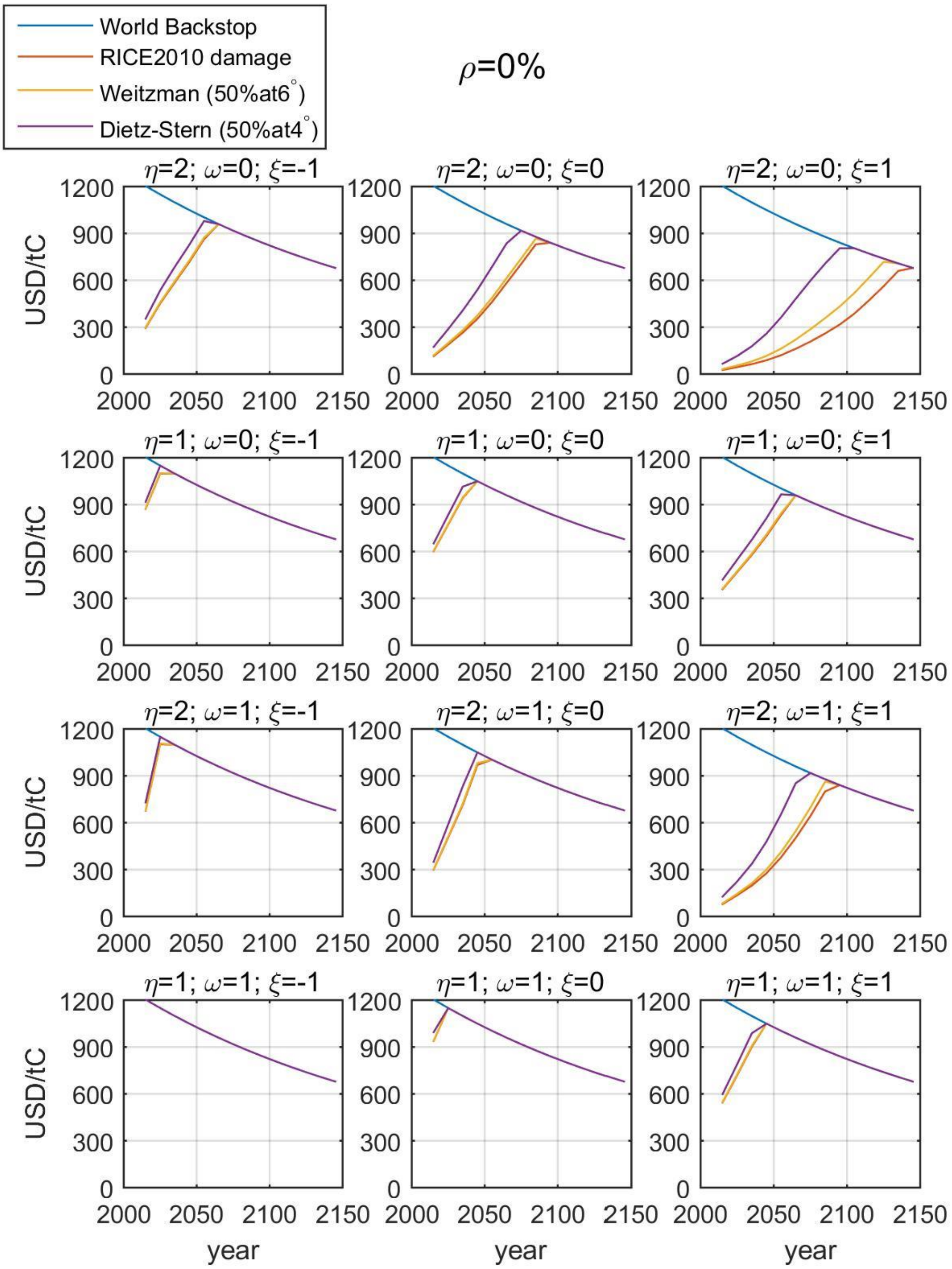

Figure 2B: Optimal carbon price paths for different damage function specifications under three different values for the income elasticity of damage, ξ, for combinations of $\eta \in \{1, 2\}$ and $\omega \in \{0,1\}$, all with $\rho = 0\%$.

References

Arrow, K. J. (1999). Discounting, morality, and gaming. in *Discounting and intergenerational equity*, (Portney and Weyant) 13-21.

Arrow, K., M. Cropper, C. Gollier, B. Groom, G. Heal, R. Newell, W. Nordhaus, R. Pindyck, W. Pizer, P. Portney, T. Sterner, R. Tol, and M. Weitzman, (2012). How Should Benefits and Costs be Discounted in an Intergenerational Context? *Resources for the Future Discussion Paper* 12-53, Washington, DC.

Burke, M., S. Hsiang, and E. Miguel, (2015). Global non-linear effect of temperature on economic production, *Nature*, **527**, 235-239.

Dasgupta, P. (2007), Commentary: the Stern Review's economics of climate change. *National Institute Economic Review.*

Dasgupta, P. (2008), Discounting climate change. *J Risk Uncertainty*, **37**, 141-169.

Dennig, F., M. Budolfson, M. Fleurbaey, A. Siebert, and R. Socolow, (2015). Inequality, Climate Impacts on the Future Poor, and Carbon Prices, PNAS, 112, 15827-15832.

Hicks, J. (1939). "The Foundations of Welfare Economics". *Economic Journal,* **49** 696–712.

Houser, T., R. Kopp, S. Hsiang, M. Delgado, A. Jina, K. Larsen, M. Mastrandrea, S. Mohan, R. Muir-Wood, D.J. Rasmussen, J. Rising, and P. Wilson, (2014), American Climate Prospectus: Economic Risks in the United States, prepared as input to the *Risky Business Project, Rhodium Group.*

Kaldor, N. (1939). "Welfare Propositions in Economics and Interpersonal Comparisons of Utility". *Economic Journal* **49,** 549–552.

Nordhaus, W., (2007), A Review of the Stern Review on the Economics of Climate Change, *Journal of Economic Literature*, **45,** 686-702.

Nordhaus, W., (2010), Economic aspects of global warming in a post-Copenhagen environment, *PNAS,* **107,** 11721-11726.

Nordhaus, W. (with P. Sztorc), (2013), User's Manual for DICE-2013R, online at: http://www.econ.yale.edu/~nordhaus/homepage/documents/DICE_Manual_103113r2.pdf

Rawls, J., (1971), A Theory of Justice, *Harvard University Press*, Cambridge, MA.

Schelling, T., (1995), Intergenerational discounting, *Energy Policy,* **23,** 395-401.

Stern, N., HM Treasury Department, UK (2006). *Stern Review on the Economics of Climate Change.*

Tol, R., (2009). The Economic Effects of Climate Change, *Journal of Economic Perspectives,* **23,** 29-51.

Weitzman, M., (2007), A Review of the Stern Review on the Economics of Climate Change, *Journal of Economic Literature,* **45,** 703-724.

Weitzman, M., (2011), Fat-Tailed Uncertainty in the Economics of Catastrophic Climate Change, *Review of Environmental Economics and Policy,* **5,** 275-292.

Weitzman M. (2012) GHG Targets as Insurance Against Catastrophic Climate Damages. *Journal of Public Economic Theory,* **14,** 221-244

Weitzman, M., (2013), Tail-Hedge Discounting and the Social Cost of Carbon, *Journal of Economic Literature,* **51,** 873-882.

World Bank (2014), World Development Indicators, Table 2.9 Distribution of income or consumption, The World Bank, Washington, D.C. online at: http://wdi.worldbank.org/table/2.9.
